# Larval toxocariasis with persistent eosinophilia in a patient with chronic hepatitis B – a case report

**DOI:** 10.1186/1471-2334-13-S1-P77

**Published:** 2013-12-16

**Authors:** Gheorghe Plăcintă, Tatiana Știrbu

**Affiliations:** 1 Department of Infectious Diseases, Faculty for Continuing Medical Education, Nicolae Testemițanu State Medical and Pharmacy University, Chişinău, Republic of Moldova

## Background

The associated forms of larval toxocariasis and hepatitis have a particular evolution with various manifestations depending on the prevalence of pathology. In both cases this liver involvement and specific antiparasitic treatment may aggravate the liver disease.

## Case report

We present the case of a patient with clinical symptoms of lightheadedness, fatigue, memory disturbances, tremor of the limbs, nausea and blurred vision. The laboratory tests found the presence of hepatomegaly, interstitial nodules on ultrasound exam, positive HBsAg, hypereosinophilia (40%) and increased titers of anti-*Toxocara* - 71.98. After diagnosing the larval toxocariasis, we prescribed treatment with albendazole and hepatoprotective drugs for 14 days. After a month, the general condition improved with the reduction of cytolysis syndrome, eosinophilia (14%) and the titer of anti-*Toxocara* (28). The patient was followed-up for 7 years, during which there were periodic relapses requiring repeated courses of benzimidazole derivatives. It is worth mentioning that the pronounced worsening of the general condition in 2010 was caused by the association of other parasitic invasions (giardiasis and ascaridiasis) which also resulted in a marked increase of eosinophilia (60%), increased titers of anti-*Toxocara* (73.47) and a high level of transaminases (ALT – 87 U/l). After treatment with albendazole the general condition improved with the disappearance of clinical signs and the reduction of cytolysis syndrome.

## Conclusion

The case shows the chronic nature of the *Toxocara* invasion with the possibility of repeated relapses and wavy evolution of the disease, with a strong correlation between the intensity of invasion and clinical picture. The presence of an association with liver pathology will only lead to worsening the evolution, as was demonstrated by the data in the literature.

